# Photoacoustic Noninvasive Blood Glucose Monitoring: A Review of Systems and Strategies for Robust Glucose Concentration Estimation, with Perspectives on Miniaturization and Wearability

**DOI:** 10.3390/s26061942

**Published:** 2026-03-19

**Authors:** Jianyu Zhang, Zhizhang Li, Min Wang, Luohan Lin, Guoxing Wang, Cheng Chen

**Affiliations:** School of Integrated Circuits (School of Information Science and Electronic Engineering), Shanghai Jiao Tong University, Shanghai 200240, China

**Keywords:** noninvasive glucose monitoring, photoacoustic, high-power pulsed laser, ultrasound transducer, analog-front-end for photoacoustic, interference suppression

## Abstract

Noninvasive blood glucose monitoring has long been a critical research focus in diabetes management. Among emerging technologies, photoacoustic sensing, combining the molecular specificity with deep penetration, has garnered significant attention. It offers rapid response and pain-free operation, making it a strong candidate for next-generation portable blood glucose monitoring devices. This review systematically traces the development and current state of photoacoustic glucose sensing, with a particular focus on the selection and optimization of core system components. It also summarizes common interference in glucose detection and outlines strategies for their mitigation, along with signal processing and signal-to-noise ratio enhancement techniques suitable for real-world applications. Addressing the growing demand for wearable continuous glucose monitors, this work analyzes the key challenges in system integration and outlines recent advances in enabling technologies. It proposes multi-technology integration approaches to bridge the gap between photoacoustic sensing and microsystem design, offering theoretical foundations and practical guidance for future research on wearable photoacoustic systems.

## 1. Introduction

Diabetes mellitus is a chronic metabolic disorder characterized by elevated blood glucose due to impaired insulin secretion or action. Major types include type 1 diabetes (T1DM), type 2 diabetes (T2DM), and gestational diabetes mellitus (GDM) [1]. These conditions disrupt glucose homeostasis and, if unmanaged, can lead to serious complications.

**Figure 1:**
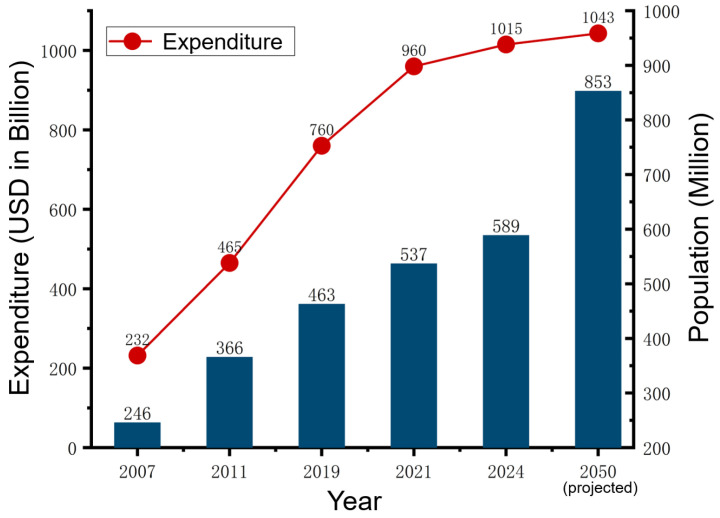
Growth in the number of people with diabetes and related medical expenditure over the years.

According to the International Diabetes Federation (IDF), diabetes contributed to an estimated 3.4 million deaths globally in 2024, with 589 million adults living with diabetes—42.8% of whom were undiagnosed, which is shown in Figure 1. The number is projected to reach 853 million by 2050, alongside an annual global healthcare cost exceeding USD 1043 billion [2]. These figures underscore the urgent need for effective, scalable monitoring and management solutions.

Given its asymptomatic onset and high complication rate, early detection and continuous monitoring are crucial, especially for T2DM, which accounts for ~90% of cases and is highly manageable in early stages. Regular health check-ups and blood glucose monitoring remain the most effective strategies for prevention and control [3].

Time in Range (TIR), defined as the percentage of time blood glucose stays within a target range (e.g., For typical T1DM and T2DM, 3.9–10 mmol/L is the target range), is now a key metric in glycemic control. Clinical guidelines recommend maintaining TIR above 70% to mitigate disease progression and complications [4]. Achieving this relies on continuous glucose monitoring combined with interventions such as dietary control, medication, and insulin therapy.

Self-monitoring of blood glucose (SMBG), based on electrochemical test strips, remains the standard for home-based monitoring due to its low cost and accuracy [5]. However, SMBG is invasive and requires frequent finger-prick sampling. As shown in Table 1, patients with T1DM, GDM, or intensive insulin-treated T2DM are advised to test over 150 times per month. For early-stage T2DM, 2–3 tests per week are recommended. Such frequent testing can lead to discomfort, infection risks, and reduced patient compliance, particularly among undiagnosed high-risk individuals, hindering early intervention. This underscores the urgent need for portable, noninvasive, continuous blood glucose monitoring devices.

To address these issues, research and development efforts toward miniaturized, noninvasive, and continuous glucose monitoring technologies have persisted. Currently, electrochemical microneedle-based Continuous Glucose Monitoring (CGM) systems have achieved commercial maturity, enabling minimally invasive continuous glucose monitoring. However, challenges such as limited sensor lifespan, invasiveness, and high consumable costs remain inherent drawbacks. Therefore, truly noninvasive CGM remains an unmet clinical need and a key research focus in biomedical sensing [8].

Over recent decades, various noninvasive physical modalities have been explored for noninvasive glucose detection, including optical, electrical, thermal, acoustic, and millimeter-wave techniques [9,10,11]. Despite encouraging results, challenges such as limited penetration depth, low specificity, and signal interference have prevented translation into real-world application scenarios.

Among emerging strategies, Photoacoustic (PA) sensing offers a promising hybrid approach by combining optical specificity with ultrasound penetration depth. Pulsed laser excitation induces localized thermoelastic expansion in tissue, generating acoustic waves whose properties are modulated by glucose-dependent optical absorption [12,13,14]. This allows indirect estimation of glucose levels. While PA sensing has been widely applied in biomedical imaging, its potential for glucose monitoring remains underexplored at the system level. Current literature lacks a focused review on system architecture, signal chain design, performance requirements, and interference mitigation in PA-based glucose sensing, creating technical barriers and limiting interdisciplinary development.

Building on the above reasons, this review traces the development and current status of PA glucose sensing, with a specific focus on PA spectroscopy-based noninvasive glucose detection, highlighting its evolving trends toward high accuracy and miniaturization. Focusing on system architecture, we analyze the time- and frequency-domain characteristics of PA signals to extract key principles for subsystem selection and integration design.

Performance requirements for critical components—such as laser sources and signal chains—are quantified, and common interference sources along with suppression strategies are discussed. Furthermore, we examine the major technological challenges currently hindering PA system integration and review the progress of enabling technologies that support miniaturization. By bridging the gap between PA glucose sensing and microsystem engineering, this review aims to offer a multidisciplinary engineering perspective and provide a theoretical and systematic foundation for developing next-generation miniaturized and wearable PA-based glucose monitoring platforms.

The core literature on the topic of photoacoustic glucose monitoring was compiled through a systematic search of the Google Scholar database (1993–2025, keywords: “blood glucose” AND “photoacoustic”). This process yielded 83 initial articles, from which 43 core publications were selected for detailed analysis after a thorough screening. For the state-of-the-art developments in key sub-modules (e.g., advanced laser sources, ultrasound transducers, and data acquisition circuits), our review is supported by high-impact publications from the leading journals, conferences, and technical literature within those respective specialized fields.

The rest of this paper is organized as follows: Section 2 reviews the development of PA glucose monitoring and analyzes the advantages and limitations compared with other blood glucose detection methods; Section 3 provides a detailed overview of the key components of photoacoustic systems and the strategies for interference suppression. Section 4 explores the miniaturization and integration possibility with emphasis on wearable PA systems for glucose detection. Finally, Section 5 concludes this paper.

## 2. Principle of Photoacoustic Sensing and Development of Photoacoustic Noninvasive Blood Glucose Detection

As described in Section 1, precise and timely blood glucose monitoring has always been central to effective diabetes management. To provide a rigorous context for the emergence of photoacoustic (PA) sensing, this section categorizes and reviews four representative monitoring approaches based on two primary criteria: (i) invasiveness (Invasive vs. Non-invasive) and (ii) physical sensing principles (Optical vs. Non-optical).

First, electrochemical detection is introduced as the “gold standard” to establish a benchmark for measurement accuracy and reliability. Subsequently, three noninvasive candidates with extensive research foundations—Infrared Spectroscopy, Raman Spectroscopy, and Metabolic Heat Conformation—are selected as representatives of long-standing exploration in the field. These methods are discussed not only to demonstrate their fundamental sensing mechanisms but also to articulate the persistent challenges that have hindered their clinical transition. By analyzing the inherent trade-offs between chemical specificity and penetration depth in these well-studied modalities—such as the high scattering limits of purely optical spectroscopy or the environmental sensitivity of thermal-based methods—we aim to highlight the scientific motivation for exploring PA techniques, which leverage both high optical contrast and high ultrasonic resolution.

### 2.1. Overview of Representative Glucose Monitoring Approaches

#### 2.1.1. Invasive: Electrochemical

**Figure 2:**
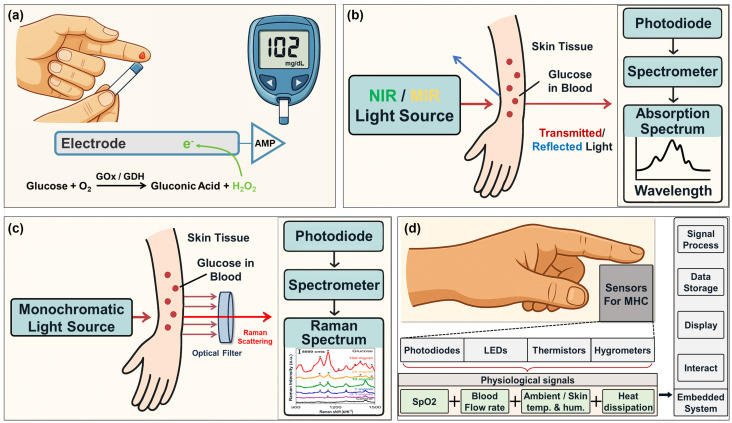
Working principles of representative glucose monitoring approaches. (**a**) Electrochemical; (**b**) Infrared spectroscopy; (**c**) Raman spectroscopy, the stars in the bottom right correspond to the characteristic peaks of the tested glucose; (**d**) Metabolic heat conformation.

Electrochemical glucose sensing remains the dominant approach for clinical and home monitoring [15], as illustrated in Figure 2a. It relies on glucose oxidase (GOx) or dehydrogenase (GDH) to catalyze glucose into hydrogen peroxide, which is oxidized at the electrode to generate a current proportional to glucose concentration [15,16]. This method offers high accuracy and rapid response, forming the basis of both SMBG and CGM systems. SMBG requires fingertip blood sampling, whereas CGM uses microneedles to continuously monitor interstitial fluid (ISF). CGM performance degrades over time as biofouling reduces enzyme activity, limiting sensor lifespan to roughly two weeks [17,18]. Mild invasiveness, consumable dependence, and wearer discomfort remain key barriers to long-term adoption.

#### 2.1.2. Noninvasive Optical Method: Infrared Spectroscopy

Infrared spectroscopy (IS), as illustrated in Figure 2b, is one of the earliest noninvasive glucose monitoring methods. It detects glucose by its characteristic wavelength absorption, with absorbance linearly related to concentration under the Lambert–Beer law [19]. Applications mainly involve near-infrared (NIR, 700–2500 nm) and mid-infrared (MIR, 2500–25,000 nm) regions. NIR monitors overtone vibrations of CH, NH, and OH bonds, offering millimeter-scale penetration and low-cost instrumentation, but it suffers from broad peaks and interference from water and lipids, requiring complex calibration [20]. MIR directly probes intrinsic glucose vibrations with higher selectivity and sensitivity, but water absorption restricts penetration to a micrometer scale. Current studies focus on the 2857–3333 nm fingerprint region using mid-infrared spectrometers to mitigate water effects, though bulkiness, cost, and integration remain key challenges [21].

To overcome the aforementioned challenges, particularly the limited penetration depth and background interference in the MIR region, several advanced approaches have been proposed. Mid-infrared fiber evanescent wave spectroscopy (MIR-FEWS), utilizing chalcogenide fibers, leverages the interaction between the evanescent field and the sample at the fiber surface. By optimizing the fiber’s waist diameter, this technique significantly enhances sensitivity, enabling simultaneous detection of glucose and triglycerides (TyG index) in whole blood with a low limit of detection (1.18 mM for glucose) and high correlation to clinical standards [22]. Alternatively, photothermal detection combined with mid-infrared quantum cascade laser (QCL) technology offers a unique solution by measuring the minute heat generated upon glucose vibrational excitation. This method probes the ISF at depths of 60−100 μm, effectively bypassing the limitations of direct optical penetration. Clinical evaluations have demonstrated its high accuracy, with over 99% of results falling within Zones A and B of the consensus error grid [23]. Despite these advances, several bottlenecks remain: MIR-FEWS sensors are currently constrained by large sample volume requirements and the risk of fiber fragility, while QCL-based photothermal devices still rely on relatively bulky light sources and are sensitive to movement artifacts during measurement. Future breakthroughs are expected to focus on the miniaturization of compact mid-infrared components and the implementation of depth-selective tomography to further enhance clinical portability and robustness.

#### 2.1.3. Noninvasive Optical Method: Raman Spectroscopy

Raman spectroscopy (RS), unlike absorption methods, uses inelastic scattering of monochromatic laser light, with shifts reflecting molecular vibrations, enabling molecular analysis [24]. Raman peaks are narrow and specific but extremely weak—only $10^{-6}$ to $10^{-10}$ of incident photons—and prone to fluorescence interference, limiting direct noninvasive glucose detection [25]. To address this, enhancement techniques like surface-enhanced Raman spectroscopy (SERS) boost signals by 6–14 orders via metal nanostructures [26]. Spatially offset Raman spectroscopy (SORS) probes glucose at the dermal–epidermal junction, achieving 14.6% mean absolute relative difference (MARD) accuracy [27]. Nonlinear methods such as Coherent Anti-Stokes Raman scattering (CARS) and stimulated Raman scattering (SRS) expand biomedical uses [28,29]. Despite advances, Raman-based glucose sensing faces challenges from weak signals, tissue scattering, and individual variability. Future work should enhance sensitivity, reduce noise, develop robust models, and create compact, portable systems for practical application [11,30,31].

#### 2.1.4. Noninvasive Non-Optical Method: Metabolic Heat Conformation

Metabolic Heat Conformation (MHC), which is shown in Figure 2d, is a multimodal noninvasive glucose sensing technique gaining attention. It relies on heat released during glucose metabolism, which affects metabolic rate, local blood flow, oxygen consumption, and heat generation, observable in skin microcirculation [32]. MHC collects multiple physiological signals—skin temperature, ambient humidity, SpO_2_, heart rate, and local heat flux—and uses multivariate models like support vector regression and neural networks to extract features and estimate glucose levels [33]. MHC offers mature sensors, simple design, low cost, and strong potential for miniaturization and wearability, suitable for smartwatches and health monitors. However, glucose–physiological signal relations show high individual variability and nonlinearity, requiring personalized modeling and long-term calibration. External factors such as temperature changes, skin conditions, motion artifacts, and pressure also affect accuracy. Developing robust, low-error glucose detection in complex, dynamic settings remains a key challenge for MHC’s practical use [11,34].

### 2.2. Fundamental and Development of Photoacoustic Noninvasive Glucose Monitoring

#### 2.2.1. Fundamentals of Photoacoustic Noninvasive Glucose Monitoring

The previously introduced IS and RS rely on single optical detection, which suffers from severe light attenuation in tissue due to absorption, scattering, and refraction—particularly in glucose-specific bands—limiting penetration to superficial regions [11,35]. In contrast, PA exploits a “light excitation–acoustic detection” mechanism, where short-pulsed laser light absorbed by glucose-containing tissue generates broadband ultrasound under thermal and stress confinement. The resulting acoustic waves are captured by high-sensitivity transducers and analyzed to quantify glucose levels [36].

**Figure 3:**
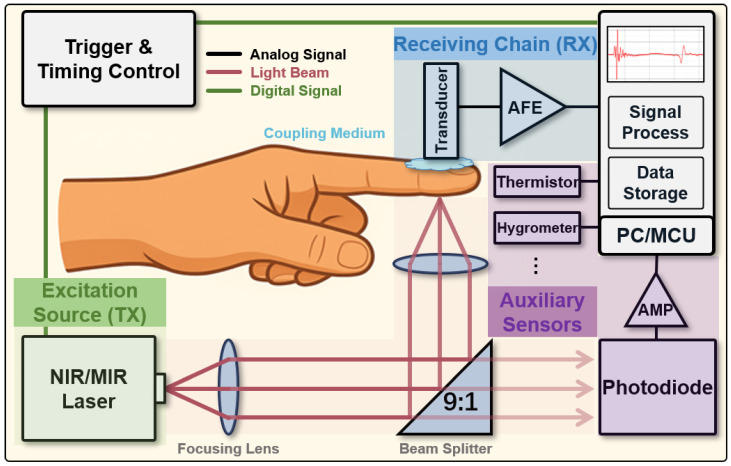
Schematic of the photoacoustic glucose monitoring system.

Through continuous refinement, system configurations for glucose-oriented PA detection have become relatively standardized. As illustrated in Figure 3, a typical setup comprises seven primary components: (1) triggering and timing control unit, (2) excitation source (TX), typically employed by NIR/MIR pulsed laser, (3) optical collimation and focusing assembly, (4) target sample, (5) PA receiving chain (RX), including a coupling medium (e.g., ultrasound gel or an integrated acoustic impedance matching layer) to ensure efficient signal transmission, an ultrasound transducer, and an Analog Front End (AFE) for PA signal detection, (6) data acquisition and processing module, (7) auxiliary sensors for noise suppression and calibration (e.g., temperature sensors, humidity sensors, and light intensity sensors such as photodiodes).

A major advantage of PA lies in its one-way detection: only optical delivery is required to generate ultrasound at depth, unlike conventional methods requiring two-way optical paths. Although PA signals are weak, tissue ultrasound attenuation (0.3–1.5 dB/cm/MHz [37]) is far lower than near-infrared light (1~20 ${\mathrm{cm}}^{-1}$ ≈ 10–90 dB/cm [38]), enabling penetration depths 3–7 times greater than purely optical approaches [39,40].

**Figure 4:**
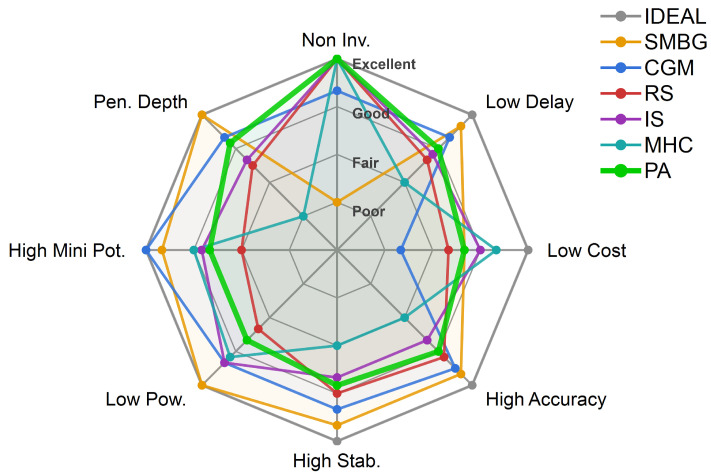
Multidimensional performance evaluation of various blood glucose monitoring techniques.

To objectively assess the strengths and limitations of different approaches, eight dimensions are considered: accuracy, delay, cost, noninvasiveness, stability, power consumption, penetration depth, and miniaturization potential. As this work does not aim to comprehensively review all glucose monitoring technologies; five representative methods are compared with PA glucose monitoring, while readers may refer to prior reviews for broader coverage [11,41,42]. The multidimensional evaluation presented in Figure 4 is formulated based on the technical parameters, performance benchmarks, and literature comparisons summarized in Table A1 of Appendix A. Although some subjectivity is unavoidable, the evaluation is guided by extensive literature to maximize objectivity.

As summarized in Figure 4, invasive methods (SMBG/CGM) remain the most accurate and stable but require consumables and retain the drawbacks of invasiveness. Among noninvasive approaches, Raman spectroscopy offers high accuracy via molecular specificity, yet its bulky and power-hungry instrumentation limits portability. MHC benefits from mature low-power hardware and miniaturization potential, but its indirect measurement is environmentally sensitive and clinically insufficient.

PA provides a unique balance. Its optical–acoustic mechanism enables millimeter-level penetration, reduced delay, and improved accuracy compared with IS through multimodal fusion. It is fully noninvasive and test-strip-free and reagent-free, lowering long-term costs, and its miniaturization potential is supported by advances in semiconductor lasers [43,44] and micro-ultrasound transducers [45,46,47]. In contrast, RS still relies on large spectrometers and ultra-sensitive detectors.

Overall, PA monitoring optimally balances the trade-offs among noninvasiveness, accuracy, and real-time performance while offering exceptional potential for miniaturization through microelectronic integration. Emerging advances, such as Application-Specific Integrated Circuit (ASIC)–Capacitive Micromachined Ultrasound Transducer (CMUT) integration and high-power Vertical-Cavity Surface-Emitting Laser (VCSEL) [48,49,50] enable ultra-compact system designs, overcoming current size and power limitations. These innovations position PA as a leading candidate for next-generation wearable glucose management.

#### 2.2.2. Development of Photoacoustic Noninvasive Glucose Monitoring

**Figure 5:**
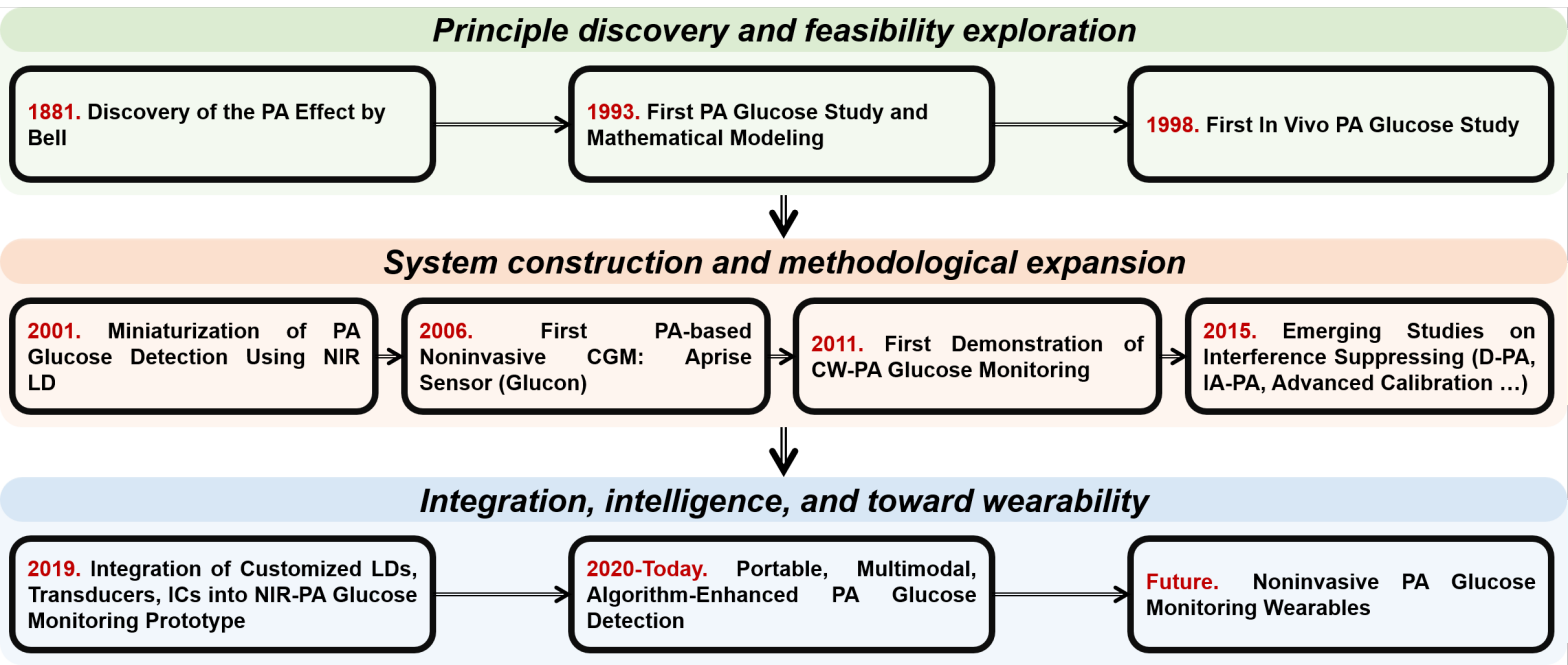
Historical development of PA glucose monitoring research until 2026 [51,57,58,59,60,61,62,63,64,65,66,67].

After establishing a fundamental understanding of PA glucose monitoring, it is important to review its historical development. As shown in Figure 5, the PA effect has been studied for over a century, originating from Bell’s 1880 photophone experiments and contemporaneous work by Tyndall and Roentgen, which first demonstrated the generation of acoustic waves via thermoelastic expansion following optical absorption [51,52,53]. Practical applications emerged in the 1930s, with Viengerov performing quantitative gas analysis using an electrostatic microphone in 1938 [54]. Subsequent advances included the Rosencwaig–Gersho solid-state model (1976) [55] and liquid-phase detection techniques by Lahmann in 1977, achieving significant sensitivity improvements [56].

A key milestone in PA glucose sensing occurred in 1993, when Christison et al. demonstrated the first measurement of glucose in whole blood using a TEA CO_2_ laser at 9676/9603 nm [57], highlighting PA’s insensitivity to optical scattering in turbid media. The same group later established a theoretical framework using a 1064 nm Nd:YAG laser and tissue phantoms, systematically analyzing thermophysical parameters and proposing multivariate strategies to enhance detection accuracy and specificity [68].

The foundational model remains relevant today: pulsed or modulated laser energy absorbed by tissue is converted to heat, inducing transient thermoelastic expansion that generates pressure waves. These waves propagate through the medium and are captured by ultrasound transducers, with the process described by the acoustic wave equation [68]:

(1) $$\left(\frac{1}{v^{2}}\frac{\partial^{2}}{\partial t^{2}}-\nabla^{2}\right)P_{a}=\frac{\alpha \beta }{C_{P}}\frac{\partial I_{a}\left(\vec{z},t\right)}{\partial t}$$

Here, $P_{a}$ is the PA pressure, α is the optical absorption coefficient, β is the thermal expansion coefficient, *v* is the speed of sound in the medium, $C_{p}$ is the specific heat capacity at constant pressure, $I_{a}$ is the instantaneous optical intensity, *t* is time, and *z* is the spatial coordinate along the beam propagation direction. By solving Equation (1), the function of the initial PA pressure can be written as:

(2) $$|P_{a}|\propto \frac{\alpha \beta \sqrt{v}}{C_{P}}$$

When the glucose concentration changes, the resulting variation in PA pressure at a specific wavelength is primarily attributed to two components: the change in the optical absorption coefficient Δα and the change in the composite physical parameter of the medium $\Delta \beta \sqrt{v}/C_{P}$. Therefore, the modified PA pressure $P_{a}^{\prime }$ after a change in glucose concentration can be expressed as:

(3) $$|P_{a}^{\prime }|\propto \left(\alpha_{0}+\Delta \alpha \right)\cdot \left[\frac{\beta_{0}\sqrt{v_{0}}}{C_{P0}}+\Delta \left(\frac{\beta \sqrt{v}}{C_{P}}\right)\right]=\left(\alpha_{0}+\Delta \alpha \right)\cdot K\cdot \frac{\beta_{0}\sqrt{v_{0}}}{C_{P0}}$$

where $K\cdot \left(\beta_{0}\sqrt{v_{0}}/C_{P0}\right)=\beta_{0}\sqrt{v_{0}}/C_{P0}+\Delta \left(\beta \sqrt{v}/C_{P}\right)$. The glucose concentration induced PA signal variation can be denoted as:

(4) $$\frac{|P_{a}^{\prime }\left|-\right|P_{a}|}{|P_{a}|}=\frac{K\cdot \alpha_{0}+K\cdot \Delta \alpha -\alpha_{0}}{\alpha_{0}}=K-1+K\cdot \frac{\Delta \alpha }{\alpha_{0}}$$

PA signal variations arise from two principal mechanisms. Elevated glucose not only modifies optical absorption but also alters other physical parameters—specific heat decreases and sound speed increases roughly linearly with concentration [37,69]. This multi-parameter response gives PA sensing higher sensitivity to subtle glucose changes than conventional spectroscopy. By selecting glucose-specific absorption wavelengths, regression models can map PA signal variations to glucose levels.

Building on PA technology’s potential for noninvasive glucose sensing, Hugh A. MacKenzie et al. conducted the first in vivo validation study using NIR photoacoustic spectroscopy (800–1200 nm) to overcome MIR water absorption limitations in 1998 [59]. Spectral analysis identified 1040 nm as optimal for glucose sensitivity. Testing eight human subjects using the Oral Glucose Tolerance Test (OGTT), their PA measurements correlated strongly with clinical blood glucose levels ($R^{2}$ > 0.84), with 91% of the data within ±20% error. This study validated in vivo glucose detection and advanced PA technology toward clinical use.

Since the early 2000s, advances in semiconductor lasers and transducers have driven PA glucose monitoring toward miniaturization. In 2001, Zuomin Zhao et al. from the University of Oulu first employed a 905 nm near-infrared pulsed laser diode (PLD) (25 W, 160 ns pulse width, 200 Hz repetition) for PA blood glucose detection [58]. The results showed a ~7% linear signal increase as glucose concentration rose from 88.2 to 500 mg/dL, demonstrating the feasibility of LD-based PA sensing and establishing a foundation for compact, low-power, portable glucometers.

Recognizing the potential of PA noninvasive blood glucose monitoring, Glucon Inc. developed the first PA-based glucose monitor (Aprise Sensor) in 2006 [60]. Testing 62 diabetic patients across OGTT, mixed-meal, and intravenous glucose tolerance tests (reference: YSI 2700, 10 min intervals), it achieved 19.9% MARD (13.2% median ARD) across 979 measurements. Clark error grid (CEG) analysis showed 94.6% in Zones A/B (66.5%/28.1%), with better MARD for OGTT (17%) vs intravenous glucose infusion (22%). However, the device exhibited several unresolved limitations, including poor sensitivity to hypoglycemia (<150 mg/dL) with ARD up to 20%, and the requirement for periodic calibration, with a 90-min calibration interval to achieve 80 mg/dL accuracy. Despite these, it pioneered noninvasive PA monitoring and inspired later miniaturized PA system improvements [70].

Traditional PA glucose detection required high-power bulky pulsed lasers, hindering miniaturization. NTT addressed this by developing low-power alternatives. In 2011, Camou et al. introduced continuous-wave PA spectroscopy (CW-PAS) using modulated DFB lasers with lock-in detection for improved SNR [61], combined with OPBS to suppress water interference [71,72]. This evolved into differential continuous-wave photoacoustic spectroscopy (DCW-PAS) (2018–2020) [73,74], using dual wavelengths (1382/1610 nm) with matched water absorption but distinct glucose sensitivity, theoretically eliminating water interference. While maintaining OPBS specificity benefits, DCW-PAS improved signal processing and practical applicability, showing strong OGTT correlation with invasive measurements. However, CW-PA still faces challenges, including limited PA cell volume, thermal sensitivity, and incomplete analyte specificity in complex physiological environments.

With rapid advances in computer science, novel calibration and regression algorithms have been increasingly applied in PA glucose research to mitigate environmental interference and improve measurement accuracy. In 2015, Pai et al. demonstrated the feasibility of PA-based CGM with a 905 nm semiconductor laser [75], later extending the study to a dual-wavelength (905 nm and 1550 nm) platform with a second-order polynomial calibration in both time and frequency domains [76]. Pai further developed a kernel-based multivariate calibration strategy to capture nonlinear PA–glucose relationships, achieving in vivo mean absolute deviation (MAD) of 10.79 mg/dL and MARD of 7.01% after individualized calibration [77]. Concurrently, Zhong Ren developed a pulsed NIR PA prototype using a tunable optical parametric oscillator (OPO) laser, introducing a least-squares calibration combined with wavelet-threshold denoising to enhance SNR [78]. In 2017, Ruochong Zhang proposed a multi-feature fusion model combining PA peak-to-peak amplitudes and time-to-peak features, providing a novel approach for improving noninvasive PA glucose prediction accuracy [79]. Collectively, these studies chart a clear trajectory of algorithmic optimization in PA glucose monitoring.

Beyond algorithmic innovations, significant progress has also been made in understanding and mitigating PA interference sources to enhance system robustness. In 2016, Jonas Kottmann systematically investigated PA interference mechanisms and introduced a fiber-coupled differential dual-QCL PA system [62]. The system utilized 1080 ${\mathrm{cm}}^{-1}$ and 1180 ${\mathrm{cm}}^{-1}$ MIR pulsed lasers and incorporated intensity normalization with differential photoacoustic PA (D-PA), suppressing physiological and environmental noise by 8× compared to single-wavelength PA. In 2018, Sim et al. combined PA spectroscopy with microscopic PA imaging (IA-PA) to investigate the influence of sweat gland secretions on noninvasive glucose monitoring [63]. The study identified sodium lactate as a key interferent in MIR photoacoustic signals. Through IA-PA-guided spatial targeting of non-glandular regions, the method achieved significant interference suppression. More recently, in 2022, Lifeng Yang analyzed external factors affecting PA signal fidelity using a 1535 nm laser and a bowl-shaped PA cell [80]. Experiments showed that laser power and temperature variations mainly alter signal amplitude, while sample–transducer spacing affects delay. A multivariate calibration model improved low-concentration glucose measurement linearity ($R^{2}$: 0.26 → 0.86). These findings advance high-accuracy, interference-resilient PA glucose sensing for noninvasive monitoring.

Over several decades of evolution, PA glucose monitoring has matured into three prominent development trends: system miniaturization, algorithm-driven signal processing, and multimodal integration. These trends have attracted broad interdisciplinary participation and injected new momentum into technological innovation.

System miniaturization has achieved key breakthroughs. Yuanjin Zheng’s team developed the first portable NIR pulsed PA glucose prototype [65]. The device integrates a 1550 nm fiber-coupled LD, a custom ultrasonic transducer, and a dedicated front-end chip, achieving a total weight of 650 g. In aqueous glucose tests, it delivered excellent performance with an $R^{2}$ of 0.89 for low concentration detection [65,66,81,82,83,84,85]. They also produced multiple PA sensing chips, addressing the large size and high cost of conventional PA acquisition hardware and accelerating the miniaturization of PA imaging and glucose monitoring.

Algorithmic optimization has seen rapid advancement. Ren and Wu have independently demonstrated the use of wavelet neural networks with particle swarm optimization and deep residual networks based on wavelet transforms, respectively, significantly enhancing single-wavelength detection capabilities and enabling high-accuracy glucose estimation under complex environmental conditions [64,86]. Xiong et al. pioneered the integration of photoacoustic time spectroscopy (PTS) with a fusion deep neural network (fDNN), developing a One-Dimensional Convolutional Neural Network with Self-Attention-Mechanism and Long Short-Term Memory Module (1D CNN–SAM–LSTM model) capable of resolving the coupled influences of temperature, glucose concentration, and laser energy. This approach demonstrated strong generalization in animal serum experiments [87].

Multimodal integration has yielded several pioneering approaches. Karlas et al. leveraged PA tomography to assess skin microvascular changes for diabetic staging [88], enabling discrimination between healthy individuals and diabetic patients via microvascular scoring. A novel time-gated PA spectroscopy technique was introduced recently [89], selectively capturing signals from specific depths (e.g., dermal microvasculature) to mitigate stratum corneum interference and enable depth-targeted glucose assessment. Swathi Padmanabhan developed polarization-enhanced PA optical rotation spectroscopy (PAPEORS) [67,90], which extracts optical rotation signatures from PA signals. By leveraging PA’s deep penetration and high spatial resolution, PAPEORS overcomes the depth limitation of traditional optical rotation and enables the detection of chiral molecules such as glucose.

Over the past decades, PA glucose monitoring has progressed beyond the proof-of-concept stage to become a well-characterized research technology with demonstrated viability in controlled studies. Through systematic research spanning laser–tissue interactions, molecular spectroscopy, and acoustic wave propagation, the field has developed comprehensive models to address key challenges such as optical excitation, ambient interference, and signal variability. Recent advances have been particularly transformative, driven by three critical developments: (1) System Architecture Innovation: including miniaturized laser sources, optimized ultrasound transducers, and integrated circuits; (2) Interference Mitigation: through advanced techniques like D-PA and spatially resolved detection to isolate glucose-specific signals; and (3) Computational Enhancements: where machine learning algorithms now enable real-time signal decoupling from complex biological matrices and discover non-linear mapping relationships. The convergence of these technological breakthroughs has transitioned PA systems from laboratory prototypes to pre-clinical implementations. Enabled by wearable-grade semiconductor lasers and AI-powered analytics, next-generation systems now consistently meet key medical device performance, demonstrating accelerated progress toward compliant noninvasive monitoring solutions.

## 3. Design and Interference Suppression in Photoacoustic Blood Glucose Monitoring Systems

In PA glucose sensing, system architecture critically impacts signal acquisition efficiency, accuracy, and the stability of glucose concentration mapping. An optimized PA detection platform requires coordinated design across light source selection, acoustic reception, signal processing, and interference suppression. Over the past decade, established techniques for PA excitation, detection, and processing have laid the foundation for reliable glucose monitoring (Figure 3). The following sections detail the design principles for three core subsystems: PA excitation, acoustic reception, and interference suppression.

### 3.1. Classification of Photoacoustic and Key Considerations for Laser Selection in Glucose Monitoring

**Figure 6:**
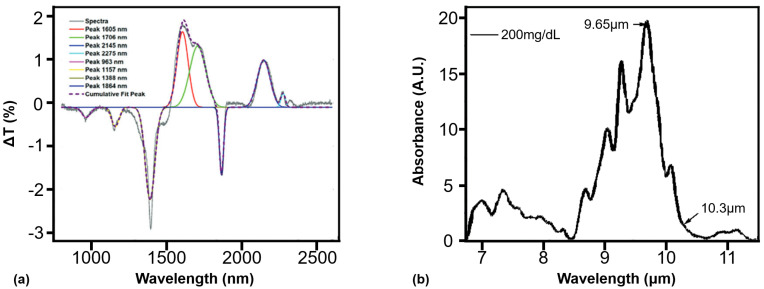
Glucose absorption features in NIR and MIR ranges. (**a**) Analysis of transmittance difference peaks between 10 g/dL glucose solution and pure water in NIR range, reprinted with permission from [92]© Wiley Advanced. (**b**) FTIR glucose absorption spectrum in the MIR range from aqueous glucose solutions with water absorption baseline subtracted, reprinted with permission from [93] © 2026 MDPI, Licensed under CC BY 4.0.

In PA-based glucose sensing, the optimization of the light source requires a comprehensive consideration of spectral matching, tissue penetration depth, and interference suppression. First, due to the inherently low photoacoustic conversion efficiency (typically <0.0001), the laser source must deliver high peak power [91]. As illustrated by the glucose absorption features in Figure 6, glucose molecules exhibit characteristic absorption peaks in both the MIR and NIR regions, necessitating the use of narrow-linewidth light sources—such as NIR wavelengths at 905 nm, 1064 nm and 1550 nm, or MIR fingerprint bands around 1034 ${\mathrm{cm}}^{-1}$ (9671 nm) and 1080 ${\mathrm{cm}}^{-1}$ (9259 nm). Among these, NIR wavelengths offer deeper penetration into biological tissue due to the relatively low water absorption coefficient, making them suitable for probing subsurface vasculature. In the MIR region, the photon energy matches the fundamental vibrational modes of glucose molecules, thereby enhancing molecular specificity; however, the strong water absorption in this band often jeopardizes the concentration resolution, posing challenges for quantitative measurements. Additionally, endogenous absorbers such as hemoglobin and lipids may exhibit overlapping absorption peaks. Therefore, it is essential to select wavelengths with strong glucose specificity to avoid spectral regions susceptible to interference from other constituents, such as hemoglobin isosbestic points.

From an excitation standpoint, PA signal generation must satisfy two key thermoelastic confinement conditions: the optical energy deposition time must be shorter than the thermal diffusion time ($t_{th}$, thermal confinement) and the stress relaxation time ($t_{s}$, stress confinement) [94]. These physical constraints require the light source to deliver pulsed energy, which can be achieved by either: (1) employing nanosecond or microsecond pulsed lasers, or (2) externally modulating a continuous-wave (CW) laser. Based on the excitation approach, PA techniques can be categorized into Pulsed PA (PPA) − offering high peak power and broadband acoustic generation − and CW-PA − providing lower average power and narrowband detection. The selection between these modes requires trade-offs among penetration depth, system complexity, and cost.

**Figure 7:**
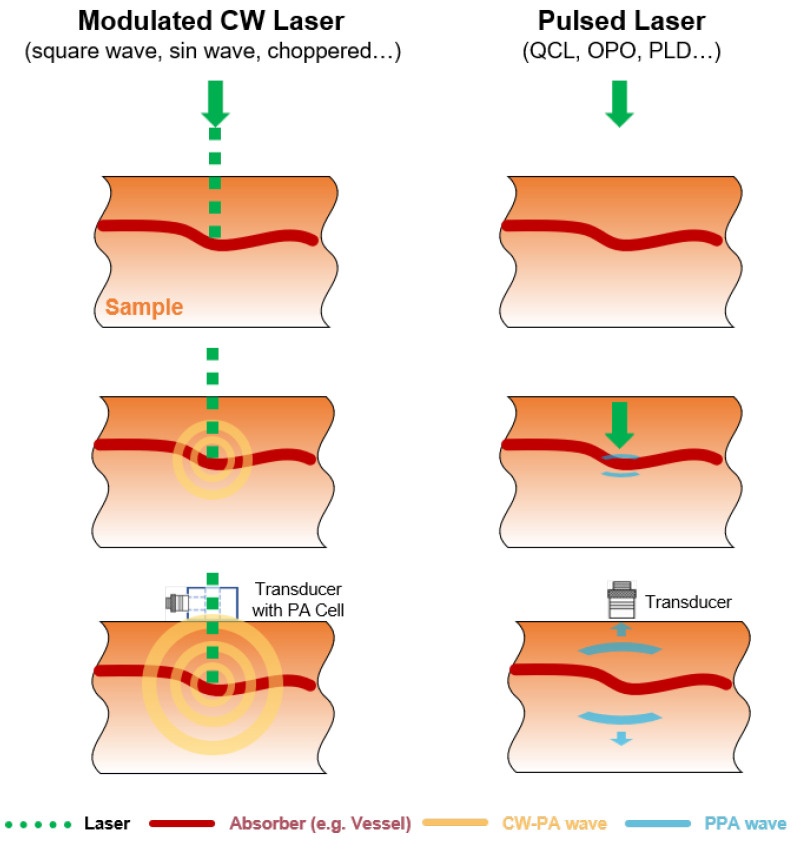
Schematic illustration of the principles of CW−PA and PPA; concept from [95].

As shown in Figure 7, PPA employs short (ns − μs) laser pulses, inherently satisfying thermal/stress confinement for instantaneous thermoelastic expansion and broadband ultrasound [94]. Compared with modulated CW sources, pulsed lasers provide distinct advantages: (1) the high peak power and narrow pulse width yield raw PA signals with high SNR facilitating separation from low-frequency environmental noise; (2) time-gating enables effective suppression of excitation noise from high-energy lasers as well as PA noise originating from the optical absorption of peripheral components; and (3) the extremely low duty cycle, despite the high peak power, minimizes thermal artifacts and reduces convection-induced perturbations [95]. In scattering media such as biological tissues, PPA amplitude reflects local absorption, while time delay correlates with depth, enabling depth-resolved imaging [47,96]. For glucose monitoring, concentration changes alter optical absorption (affecting amplitude) and acoustic velocity (reducing propagation delay) [78,97], making peak-to-peak amplitude and time-to-peak key quantification features.

CW-PA and PPA exhibit fundamental differences in excitation and signal properties. CW-PA employs continuous laser irradiation with periodic energy deposition via amplitude or frequency modulation, producing narrowband acoustic signals at the modulation frequency and harmonics [55,98]. When the modulation period is shorter than thermal diffusion and stress relaxation times, efficient PA conversion is maintained, enabling material detection applications. Modulation is typically achieved through: (1) built-in TTL triggering (square/sine-wave modulation) or (2) external optical choppers for high-power lasers lacking electronic modulation. However, chopper mechanical stability critically impacts beam quality and detection accuracy [99,100]. As shown in Figure 7, modulated CW excitation generates a propagating “thermal wave” from periodic energy diffusion in tissue [101]. Compared to PPA, CW-PA suffers from lower PA conversion efficiency and poorer initial SNR due to longer pulse widths. Yet, its narrowband spectrum permits acoustic resonance enhancement in PA cells. Combined with lock-in amplification, this enables high-SNR signal recovery [73,74,102], facilitating advancements in glucose monitoring.

Although PPA and CW-PA differ in their operating principles, the determination of laser power parameters in both cases can be systematically derived by first considering the PA excitation constraints, followed by incorporating biosafety requirements and laser energy utilization efficiency. The procedure is as follows. First, the laser pulse width $t_{pulse}$ should satisfy both the thermal and stress confinement conditions. The thermal confinement condition requires that $t_{pulse}$ be shorter than the thermal diffusion time following optical energy absorption, given by $t_{th}\approx {L_{P}}^{2}/D_{T}$, where $L_{P}$ is the characteristic dimension of the irradiated tissue and $D_{T}$ is the thermal diffusivity of the sample. The stress confinement condition requires $t_{pulse}$ to be shorter than the acoustic transit time across the optically absorbing region, expressed as $t_{s}=L_{P}/v$[94], where *v* is the acoustic velocity in tissue. For example, with v≈ 1500 m/s, $D_{T}\approx$ 1.4 × $10^{-2}$ ${\mathrm{cm}}^{-2}$/s, and $L_{P}$ = 150 μm in soft tissue, one obtains $t_{th}\approx$ 160 μs and $t_{s}\approx$ 100 ns. In this case, setting the laser pulse width below 100 ns ensures effective PA excitation.

Once the pulse width is determined, the laser energy and power must be further optimized. In theory, higher single-pulse energy improves the SNR of the PA signal and reduces the difficulty of downstream signal detection [47]; however, strict compliance with laser safety standards is essential to prevent tissue damage. The key safety metric is the maximum permissible exposure (MPE), calculated according to the American National Standard ANSI Z136.1–2022 [103], with thresholds specified for different wavelengths and pulse widths. For instance, for pulse widths of between 1 ns and 100 ns and wavelengths in the 400–1400 nm range, the MPE for skin exposure is given by:

(5) $$\mathrm{MPE}=2\cdot C_{A}\cdot 10^{-2}\frac{J}{{\mathrm{cm}}^{2}}$$

where $C_{A}$ is a wavelength-dependent correction factor: $C_{A}=1$ for 400–700 nm; $C_{A}=10^{0.002\lambda -700}$ for 700–1050 nm; and $C_{A}=5$ for 1050–1400 nm. For longer wavelengths within the same pulse duration, the MPE limits are further specified to prevent skin damage: the MPE is 0.3 J/${\mathrm{cm}}^{2}$ for 1400–1500 nm, 1.0 J/${\mathrm{cm}}^{2}$ for 1500–1800 nm, 0.1 J/${\mathrm{cm}}^{2}$ for 1800–2600 nm, and decreases to 0.01 J/${\mathrm{cm}}^{2}$ for the 2600 nm–1000 μm range. By computing the MPE and incorporating the actual beam-shaping efficiency and spot size, the maximum allowable laser power can be estimated, enabling selection of the optimal excitation energy within the safety limits.

The choice of light source in PA glucose detection systems primarily involves two major categories: solid-state lasers and semiconductor lasers. Solid-state lasers, particularly Q-switched Nd: YAG, serve as laboratory standards for fundamental studies (e.g., glucose−PA signal relationships, resonance theory) due to their high energy output (10 ns pulse width, 10 Hz repetition rate, 10 mJ/pulse) and beam stability (<5%) [62,104]. Diode pumping can boost repetition rates to hundreds of Hz, enhancing average power and reducing acquisition time [95]. OPO lasers further enable multi-wavelength PA spectroscopy through tunability [67,90,105], though their bulkiness and cost limit clinical use. The advent of MIR high-power QCL has overcome the penetration depth limitations of biological tissues, thereby advancing in vivo PA glucose research and enabling the successful acquisition of multi-site PA data [62,106,107]. However, such systems still face notable challenges, including the need for professional operation due to laser exposure hazards and low levels of system integration.

In contrast, the rapid development of LD technology in recent years has demonstrated significant advantages—compact size, high electro-optical conversion efficiency, low cost, and excellent reliability—making it an ideal candidate for portable systems [65,108,109]. Current limitations include low peak power (<100 W), necessitating driving electronics optimization (e.g., pulse stacking) and thermal management. Large beam divergence (40°) also demands beam-shaping optics. Overcoming these challenges is critical for portable PA glucose monitoring.

### 3.2. Photoacoustic Transducer and Analog Front-End Circuit for Glucose Monitoring

Photoacoustic signal generation in target tissue is only the first step; subsequent reception, amplification, and processing determine a glucose monitoring system’s quantitative accuracy and dynamic range. Ultrasound transducer selection, AFE design, and digitization circuits are critical for high-precision measurements. This subsection analyzes the PA receiving chain, quantifying module performance requirements to guide receiver design for PA glucose detection.

#### 3.2.1. Consideration of PA Transducer

In noninvasive PA glucose monitoring, the ultrasound transducer—which converts PA mechanical waves into electrical signals—is the core component. Its performance directly determines weak-signal detection capability and glucose measurement precision. Due to laser safety limits, PA signal amplitudes are often three orders of magnitude lower than in medical ultrasound imaging [110]. Additionally, nanosecond-laser-excited PA signals exhibit broadband characteristics (hundreds of kHz to hundreds of MHz), demanding specific transducer requirements for bandwidth, center frequency, sensitivity, size, and acoustic impedance.

Effective PA signal detection typically requires a transducer center frequency >1 MHz and a frequency response spanning tens of MHz [67,75,78,90,111]. Notably, frequency selection depends on application scenarios: higher frequencies attenuate more in biological tissue, necessitating lower center frequencies and narrower bandwidths for deeper layers [112].

The detection sensitivity of a transducer, expressed in mV/Pa or V/Pa, represents its ability to convert acoustic waves into electrical signals. For PA glucose detection with 1 mJ/${\mathrm{cm}}^{2}$ optical energy, signal amplitudes typically range from several to tens of pascals, further reduced by propagation distance and low absorption coefficients. To maintain detectable signals in the hundreds of µV to mV range, transducer sensitivity typically exceed 1 mV/Pa [62,99,113]. This key metric, determined by material properties (piezoelectric coefficient, structural response) and aperture area, directly impacts weak-signal detection. Larger apertures (>10 mm diameter) accumulate more acoustic energy, generating higher output voltages and improving low-amplitude signal perception [104,111].

(6) $$R={\left(\frac{Z_{2}-Z_{1}}{Z_{2}+Z_{1}}\right)}^{2}$$

Here, $Z_{1}$ and $Z_{2}$ represent the acoustic impedances of the two media. Equation (6) indicates that the poorer the impedance matching, the higher the reflection coefficient and the lower the acoustic energy transmission efficiency. Table 2 lists the acoustic impedances of typical materials. For example, when an acoustic wave propagates from skin into air, the reflection coefficient is approximately 0.99, meaning that only 1% of the mechanical wave energy can be effectively detected. Therefore, in practical PA system design, matching layers or coupling media (e.g., ultrasound hydrogel) are commonly introduced to buffer the impedance mismatch, optimize acoustic energy coupling efficiency, and enhance the effective receiving sensitivity.

In PA glucose monitoring, piezoelectric transducers remain the most established reception solution. Common materials include PZT (lead zirconate titanate) ceramics and PVDF (polyvinylidene fluoride) polymers, each with distinct advantages. PZT offers superior piezoelectric performance and conversion efficiency for high-sensitivity systems, yet its high acoustic impedance (~30−35 MRayl), tissue mismatch, and rigidity hinder wearable integration [115]. PVDF’s lower impedance (2−4 MRayl) better matches soft tissue while providing flexibility and biocompatibility, making it ideal for wearable systems [116]. However, PVDF’s lower piezoelectric coefficient yields weaker output and reduced stability versus PZT. Transducer selection thus requires balancing sensitivity, flexibility, and impedance matching for optimal performance.

#### 3.2.2. Consideration of PA AFE

**Figure 8:**
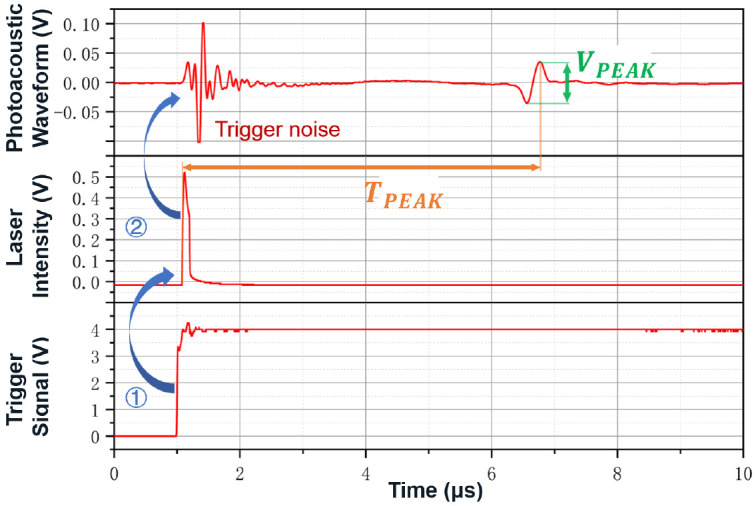
Typical PA waveform. The blue arrows and serial numbers indicate the excitation relationship, green arrows represent $T_{peak}$, and yellow arrows represent $T_{peak}$.

In the PA receiving chain, the AFE critically determines overall sensitivity and SNR following the transducer. Figure 8 shows a typical pulsed PA waveform: the trigger signal controls the laser to emit a pulse. The receiving end first detects strong electromagnetic interference (EMI), and after a certain delay, the PA signal is received. The signal’s spectral characteristics depend on laser pulse width, medium properties, and transducer response, with a bandwidth ≈ 2.35 × the pulse width [76]. Given low conversion efficiency, received PA signals typically range in microvolts, further attenuated in noninvasive/deep-tissue applications [64,117,118]. The AFE must thus provide low-noise, wideband amplification with bandpass filtering to suppress EMI and baseline drift, enhancing dynamic range while preventing saturation and ensuring clean signals for digitization.

To meet these requirements, typical pulsed PA detection systems employ a combined “filter + amplification” strategy [65,119,120,121]. A passive bandpass filter, matched to the center frequency of the transducer, is first inserted at the transducer port to suppress out-of-band noise and EMI. The signal is then amplified using either a low-noise amplifier (LNA) or a charge amplifier. LNAs offer low input noise (typically <2 nV/$\sqrt{\mathrm{Hz}}$) and moderate gain (∼25 dB), making them suitable for applications with low excitation energy and limited initial SNR [75,76,120]. Charge amplifiers, on the other hand, are preferred for charge-output-type sensors and offer high sensitivity (∼10 V/pC) and excellent EMI immunity [97,104,122,123]. An optional second-stage amplifier, typically a programmable gain amplifier (PGA), may then be used to further increase the signal level. Overall, the total gain of the RX is generally kept within 30−60 dB to ensure that the output voltage is well-suited to the input range of downstream digitization devices such as analog-to-digital converters (ADCs), data acquisition cards (DAQs), or oscilloscopes [64,65,76,105,111,120]. It is generally recommended to adjust the signal amplitude to approximately 50% of the ADC’s full-scale range to balance dynamic range and resolution.

In contrast, for CW-PA detection, the choice of front-end amplifier is more fixed. In CW-PA systems, the laser is modulated at a specific frequency (typically tens of kHz) to excite a modulated PA signal in the target medium. As CW-PA signals are even weaker than pulsed PA signals and more susceptible to broadband noise, a lock-in amplifier (LIA) is often employed for coherent detection and amplification. The reference clock of the LIA is synchronized to the laser modulation clock, and the input PA signal is internally mixed to extract the frequency-coherent PA component. A suitable time constant is then applied for low-pass filtering, effectively suppressing out-of-band noise and enabling highly selective amplification of the modulated PA signal [73,74,102].

The final digitization of the PA signal is performed by an Analog-to-Digital Converter (ADC), whose voltage and temporal quantization precision are of decisive importance in the signal chain. As noted earlier, two key PA signal features —peak-to-peak amplitude and time-to-peak—are the most commonly used metrics for quantifying blood glucose concentration changes. This means that the limiting factor for quantization precision is not the absolute values of these features but rather the changes induced by glucose concentration variation: $\Delta V_{peak}$ and $\Delta T_{peak}$. Experimental studies have shown that in a typical PA glucose detection system (λ = 1535 nm, pulse width = 4 ns, single-pulse energy = 365 μJ, transducer center frequency = 2.25 MHz, total gain = 30 dB), a 1 mg/dL change in glucose concentration induces a fluctuation of approximately 5.32 μV in the PA peak-to-peak amplitude [80]. Thus, to achieve ±15 mg/dL resolution, the system must have a voltage resolution of about 80 μV. Assuming a full-scale range of ±1 V, this requires at least 15 effective number of bits (ENOBs) in the ADC to avoid quantization noise overshadowing the signal.

Similarly, small changes in the acoustic velocity also cause slight shifts in PA signal arrival time. Given that the typical acoustic velocity in tissue is about 1500 m/s, an increase in glucose concentration leads to an increase in velocity. Studies have shown that a 1 mg/dL change in glucose causes a velocity variation of 0.0037 m/s [97]. For a typical propagation distance of 10 mm (distance between the transducer and PA source), this corresponds to a delay change of about 160 ps/mg/dL. To resolve a 15 mg/dL glucose difference, the system must discern a peak time shift of 2.4 ns, which in principle requires a sampling rate of at least twice this resolution (≥0.83 GS/s). In practice, however, high-speed acquisition devices with ≥0.2 GS/s sampling rates are often sufficient, as anti-aliasing margins and curve-fitting accuracy can be compensated through data averaging-induced temporal resolution enhancement [67,87,105,118,121,124].

It should be noted that the above voltage and temporal resolution requirements are theoretical estimates based on a representative PA system. In practical applications, the overall performance of the signal chain should be optimized through system-level co-design. Strategies such as increasing laser excitation energy, selecting high-sensitivity transducers, optimizing acoustic coupling interfaces (e.g., impedance matching layers), designing appropriate preamplifier gain and filtering schemes, and implementing effective noise suppression algorithms can all enhance signal quality and SNR, thereby relaxing the extreme requirements on backend ADC resolution and sampling rate.

**Figure 9:**
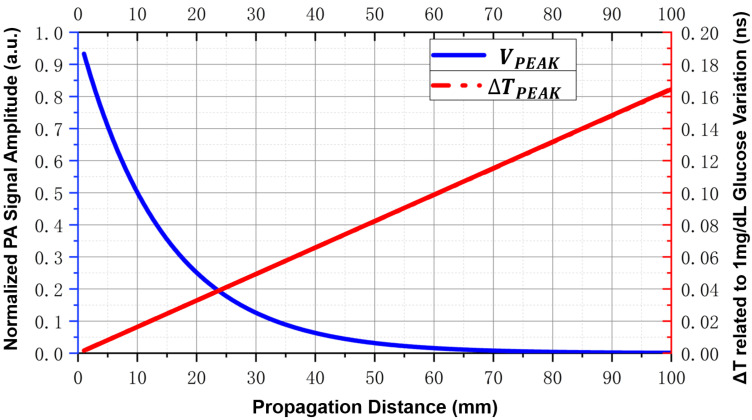
Trade-off between time-delay sensitivity and PA signal amplitude.

For the extraction of the feature time-to-peak, it is also necessary to rationally design the PA signal propagation path. Figure 9 illustrates the trade-off between time-delay sensitivity and PA signal amplitude at the detection position. A typical ultrasound attenuation model is adopted:

(7) $$A\left(d,f\right)=A_{0}\cdot 10^{-\alpha (f)\cdot \frac{d}{20}}$$

in which A(d,f) denotes the raw PA signal amplitude received at the transducer, $A_{0}$ is the initial PA source amplitude, d is the propagation distance, and α(f) represents the acoustic attenuation coefficient in the medium at frequency *f* (in dB/cm/MHz) [37]. Assuming the propagation medium is muscle tissue, the attenuation coefficient at 5 MHz can be estimated as α≈5× 1.2 dB/cm/MHz. As shown in Figure 9, longer propagation distances (e.g., 100 mm) can significantly amplify the change in $\Delta T_{peak}$ corresponding to a unit variation in sound velocity, thereby relaxing the requirements for time resolution of the receiver. However, this comes at the cost of more severe signal attenuation, making peak localization more susceptible to noise. Conversely, shorter propagation distances (e.g., 1 mm) can improve signal strength and instantaneous SNR, but $\Delta T_{peak}$ will shrink to the picosecond scale, imposing nearly impractical requirements on the sampling rate. Therefore, the propagation path design should balance observability and time-resolution requirements to meet both peak detection and delay estimation accuracy under practical engineering constraints.

In recent years, substantial progress has been made in the chip-scale integration of PA sensing, with continuous improvements in sensitivity, integration density, and energy efficiency. However, to date, no dedicated integrated circuit design targeting PA-based glucose detection has been systematically reported in the open literature. Given that glucose monitoring is highly sensitive to extremely small signal variations, the realization of a PA-based glucose monitoring system still heavily relies on high-performance analog front-ends and high-precision ADCs to ensure the reliability and integrity of raw signal acquisition. Consequently, researchers often employ algorithm-level compensation to further enhance the system SNR, including techniques such as large-scale data averaging, model-based adaptive filtering, and covariance-based noise reduction. This “front-end hardware enhancement + back-end algorithm optimization” co-design approach has become an essential strategy to ensure the feasibility of PA glucose monitoring at the current stage.

### 3.3. Interference Mechanisms and Suppression Methods in Photoacoustic Glucose Monitoring

**Figure 10:**
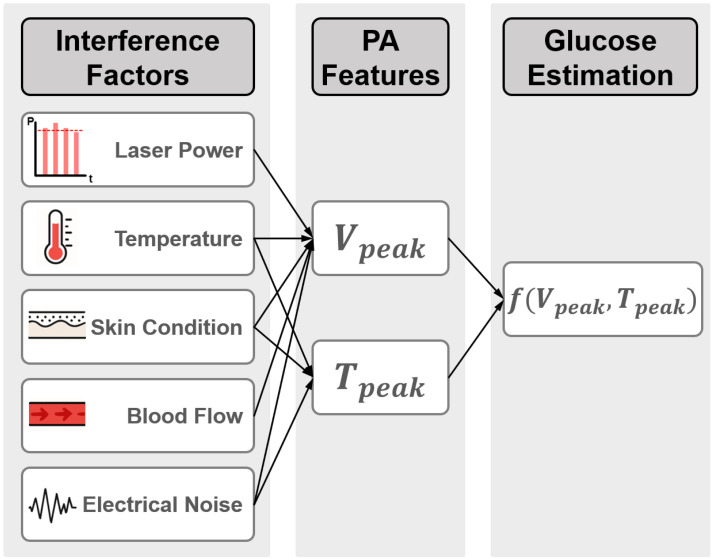
The relation between interference factors and PA features.

Despite the potential of portable PA noninvasive glucose monitoring, its clinical translation has been hindered by inaccuracies stemming from unmitigated interference across the entire signal chain—from acoustic generation to final modeling. Therefore, comprehensive understanding and effective suppression of these interference factors are critical. As discussed previously, the initial PA pressure is intrinsically linked to a series of physical parameters. To better illustrate the potential sources of interference, Equation (2) can be reformulated into Equation (8),

(8) $$P_{0}=\eta_{th}\cdot \Gamma \cdot A_{e}$$

where the Grüeneisen parameter is given as $\Gamma =\beta \cdot v^{2}/C_{P}$, $A_{e}$ denotes the specific optical absorption, and $\eta_{th}$ is the percentage that is converted into heat [94]. As indicated in Equation (8), in PA glucose studies, the PA signal is actually influenced by two factors: First, glucose concentration induces linear variations in both Γ and *v*, modulating both the amplitude and the propagation delay of the PA signal directly. Second, in practical measurements, the signal is distorted by multi-source interferences, including instrumental noise, environmental fluctuations, and biological tissue heterogeneity, as displayed in Figure 10. These factors distort the intrinsic relationship between signal features and true glucose levels, which largely explains the inconsistent results reported in the existing literature.

As noted earlier, the linear dependence of Γ and *v* on glucose concentration at the signal-feature level provided the rationale for using peak-to-peak amplitude and time-to-peak delay in glucose estimation. With further research, additional features have been explored, including time-domain metrics (e.g., logarithmic slope of the rising edge, integrated waveform area), frequency-domain metrics (e.g., spectral resonance peaks, spectral energy), and time-frequency features (e.g., Hilbert transform envelope). To mitigate random noise, techniques such as signal averaging and wavelet denoising are commonly employed [64,68,77,89,117]. Nevertheless, such preprocessing methods cannot fully eliminate systematic interferences. For instance, fluctuations in laser pulse energy directly alter the initial acoustic pressure; variations in tissue microstructure modulate propagation characteristics; and physiological factors such as body temperature or blood flow velocity can mask glucose-induced changes. These interferences rarely occur in isolation but are often coupled, forming a complex disturbance network. As a result, traditional univariate correction approaches offer only limited effectiveness.

Consequently, contemporary research in PA-based glucose monitoring is shifting from passive suppression toward active decoupling. On the one hand, carefully designed experimental strategies—such as differential detection schemes and environmental stabilization—are employed to minimize interference intensity at the source. On the other hand, advanced modeling strategies leverage multimodal data fusion and adaptive algorithms, including physics-constrained machine learning methods, to achieve interference compensation during signal interpretation. The following section will elaborate on the underlying mechanisms of these interference sources and summarize representative suppression methodologies, providing guidance for building a more robust and clinically viable PA glucose detection framework.

#### 3.3.1. Laser Source Fluctuations

In PA glucose detection systems, the stability of the laser source is one of the decisive factors affecting measurement accuracy. Solid-state lasers, such as Nd: YAG lasers, are widely employed due to their high pulse energy and excellent monochromaticity. Nevertheless, their output intensity inevitably exhibits fluctuations, which originate primarily from two sources. First, inherent physical characteristics of the laser—such as pump-source instability, thermal lensing, and thermal drift of optical components—can result in pulse energy variations as high as 3–5% at the factory specification stage. Second, during long-term operation, factors including temperature fluctuations, supply voltage instability, optical component degradation, and temporal drift further exacerbate output instability.

According to Equation (8), fluctuations in laser energy directly alter the absorbed optical energy density $E_{0}$, thereby affecting the initial acoustic pressure amplitude $P_{0}$. Experimental investigations have demonstrated that, in PA glucose detection using aqueous glucose solutions, a fluctuation as small as 0.01 μJ in laser energy can induce measurable changes in PA amplitude, corresponding to a glucose concentration deviation of up to 20.8 mg/dL [80]. This error exceeds the clinically acceptable threshold (typically <15 mg/dL), highlighting the necessity of effectively suppressing laser-induced interference.

To mitigate the impact of laser fluctuations, two primary strategies have been adopted. The first is signal averaging, wherein a large number of PA waveforms are acquired and averaged to statistically suppress random noise [63,68,77,89,117]. The second commonly used approach is real-time optical intensity monitoring with normalization, in which the laser output is split, and a photodiode is employed to monitor optical intensity continuously. During data processing, the corresponding PA signals are normalized to compensate for intensity variations. Given the linear proportionality between PA amplitude and laser energy ($P_{0}\propto E_{0}$), this method enables effective compensation of intensity fluctuations [57,63,72,80,125]. Despite their utility, these methods still face inherent limitations. Signal averaging cannot eliminate systematic bias and inevitably sacrifices temporal resolution. Beam splitting reduces laser-to-acoustic conversion efficiency and may introduce additional optical errors, while the assumption of linear proportionality may not strictly hold in heterogeneous biological tissues. Accordingly, future studies may explore more robust correction strategies, such as adaptive filtering approaches or machine learning-based nonlinear compensation algorithms, to further enhance the reliability of PA glucose detection under laser intensity fluctuations.

#### 3.3.2. Temperature Fluctuations

Temperature effects critically interfere with PA glucose detection through multi-scale mechanisms [97,99,105,123,126]. At the molecular level, temperature alters biological media properties: (1) water’s thermodynamic parameters (thermal expansion, acoustic velocity, specific heat), contributing to parameter Γ in Equation (8), nonlinearly affect PA signal amplitude/delay [126]; (2) tissue optical properties, especially water’s NIR absorption, exhibit temperature dependence [62,97]. Prakash et al. noted strong spectral sensitivity (900−1900 nm) and a “silent point” near 4 °C where thermal expansion nears zero, aiding compensation [126]. Physiologically, human temperature fluctuations (±0.5 °C) cause significant errors. Quantitative studies by Linfeng Yang have demonstrated that, at 1535 nm, a temperature drift as small as 0.01 °C results in a PA signal bias equivalent to 12.35 mg/dL glucose concentration, a value approaching the clinically acceptable error threshold [80]. In vivo, spatial temperature gradients further couple with glucose signals. Systematically, environmental shifts also introduce instrumental baseline drift [127].

To address temperature-induced interference, researchers have proposed two primary solution categories: active temperature control and passive compensation.

Active temperature control aims to stabilize the measurement environment via precision regulation. Prakash et al. developed a temperature-controlled mid-infrared PA spectroscopy system using semiconductor cooling elements and six thermocouples in a closed-loop configuration, combined with mechanical stirring to ensure uniformity. This design achieved temperature stability within ±0.1 °C [126]. Xu et al. further optimized this approach by employing a gold-coated copper PA cell and a thermoelectric cooler (TEC), reducing deviations below 0.02 °C and significantly enhancing measurement stability [128]. Camou et al. implemented a water-bath control system, leveraging a 30 L water bath with surface plastic spheres to minimize evaporation-driven fluctuations. Their design reduced temperature error from ±0.2 °C to ±0.05 °C, thereby improving glucose detection accuracy from ±26 mg/dL to ±11 mg/dL [129].

Passive compensation approaches, in contrast, mitigate temperature-related artifacts through algorithmic correction or sensor calibration [80,99,104,105]. For instance, Zhao integrated body temperature monitoring with PA signal acquisition, establishing a temperature–signal correction model to compensate for thermal interference [97]. In 2018, Tao et al. conducted an in-depth investigation into the influence of temperature on PA signals, demonstrating that the PA temperature coefficient (TC) (V/°C) depends solely on the incident light intensity and remains unaffected by variations in glucose concentration. Under their experimental conditions, the measured TC of the was found to increase with higher excitation light intensity. Specifically, as the incident light intensity rose from 1.61 V to 3.49 V, the corresponding temperature coefficient increased from 0.046 V/°C to 0.13 V/°C. These findings further validate the effectiveness of temperature compensation [123]. In addition, differential detection techniques have also been applied, wherein reference signals from standard samples are simultaneously recorded to eliminate baseline drifts induced by temperature fluctuations and other environmental noise sources [62,104,125,130].

It is noteworthy that the choice of temperature management strategy must be tailored to the application scenario. For in vitro systems, active thermal control typically provides superior performance. In contrast, in vivo monitoring requires hybrid strategies that integrate real-time body temperature tracking with algorithmic compensation. Looking forward, future research may explore the synergistic integration of intelligent temperature-compensation algorithms with miniaturized thermal-control modules, enabling precise thermal management in PA-based glucose monitoring under practical physiological conditions.

#### 3.3.3. Physiological Properties

The accuracy of PA glucose detection is not only affected by external environmental factors but also significantly influenced by the physiological properties of the target medium itself. As the primary detection interface, the skin produces secretions that interfere with PA measurements. Specifically, sweat (containing water, electrolytes, and sodium lactate) and sebum (composed of lipids and waxes) exhibit pronounced absorption peaks in the NIR region, which spectrally overlap with glucose-specific absorption bands [63]. The situation is further complicated by the anatomical heterogeneity of skin tissues. For example, sweat gland density on the fingertip (~350 glands/${\mathrm{cm}}^{2}$) is more than threefold higher than that on the forearm (~108 glands/${\mathrm{cm}}^{2}$). In contrast, sebaceous glands are highly concentrated in the facial T-zone (~400/${\mathrm{cm}}^{2}$) but are nearly absent on the palm. Such anatomical disparities result in site-dependent variations in PA background signals, which in turn affect measurement consistency [12].

To address these challenges, researchers have explored multiple strategies. Early studies limited the data acquisition period (<2 h) to minimize the impact of time-dependent physiological variations in skin secretions [58,131]. Subsequent approaches introduced nitrogen gas flow into the measurement chamber, stabilizing the local microenvironment and reducing errors associated with sweat evaporation and water condensation [62,99,132]. Moreover, differential PA techniques, similar to those employed in temperature interference suppression, have been applied. Single-wavelength differential methods using reference samples can partially compensate for skin variability [67,89,104,125], but dual/multi-wavelength differential strategies have proven more effective. These methods employ a glucose-insensitive reference wavelength alongside a glucose-sensitive primary wavelength to isolate glucose-specific signals [73,74,104,133]. For instance, Tanaka et al. developed the DCW-PAS approach for glucose monitoring. In their system, dual-wavelength excitation at 1382 nm and 1610 nm was employed—wavelengths with identical water absorption but differential glucose absorption. Comparative experiments against three invasive methods (flash glucose monitoring, self-monitoring blood glucose meters, and venous blood sampling) demonstrated good temporal consistency, with correlation coefficients ranging from 0.5 to 0.80 [73]. In addition, Sim et al. developed a multimodal system that integrated PA imaging with PA-based glucose detection. By using imaging to avoid regions with dense sweat gland distribution, they significantly reduced sweat-induced interference, achieving a MAD of 18.51 ± 12.35 mg/dL [63].

#### 3.3.4. Blood Flow Hemodynamics

In PA glucose detection systems, blood flow hemodynamics constitute another factor affecting measurement accuracy. Studies have shown that variations in blood flow velocity can significantly influence the generation and propagation of PA signals by altering the spatial distribution of red blood cells (RBCs). In 2001, Zuomin Zhao and colleagues first elucidated this phenomenon: when blood flow velocity is low, insufficient shear force promotes RBC aggregation, leading to the formation of plasma gaps. This microstructural change results in two critical optical effects: a pronounced decrease in the scattering coefficient, thereby increasing light penetration depth, and a reduction in the effective optical absorption area, which reconstructs the distribution of acoustic sources and ultimately lowers PA signal amplitude [58]. To address this challenge, Zhao demonstrated in ex vivo experiments that setting the flow velocity of a roller pump to a low but constant value effectively reduces the impact of RBC aggregation on PA signals. However, direct control of blood flow in vivo remains technically infeasible, thus shifting research efforts toward mathematical modeling for inverse compensation. For example, in 2020, Zhong Ren and colleagues in China developed a back-propagation neural network model that incorporated blood flow velocity and other physiological parameters to establish nonlinear mappings with PA peak-to-peak values and glucose concentration, thereby partially compensating for the confounding effects of flow velocity [134].

The current state of research suggests that investigations into blood flow velocity effects are still underdeveloped compared with other interference factors. Future work should focus on: (1) Developing miniaturized blood flow monitoring techniques, such as integrated modules based on Doppler ultrasound or photoplethysmography (PPG), to support real-time flow calibration; (2) Establishing quantitative models of RBC aggregation dynamics to refine mathematical descriptions; and (3) Optimizing multimodal data fusion algorithms to improve interference suppression. These advances will enable more stable and reliable in vivo PA glucose monitoring systems, thereby laying the groundwork for clinical translation. Importantly, with the advent of wearable technologies, addressing the challenge of reconciling motion-induced blood flow fluctuations with measurement accuracy may emerge as a new research frontier.

#### 3.3.5. Advanced Signal Processing: From SNR Enhancement to Machine Learning-Based Regression

Beyond mitigating specific interference sources, advanced data acquisition and processing techniques play a pivotal role in improving the SNR of PA glucose detection. Signal preprocessing typically involves three steps: (i) Mean centering to eliminate systematic offsets, (ii) Employing polynomial smoothing or a bandpass filter function to suppress out-of-band noise, and (iii) First- or second-order derivative transformations to remove baseline drifts [135]. To quantitatively characterize system noise, Allan variance analysis is commonly employed to assess temporal stability [136]. Given the inherently low SNR of PA signals, signal averaging remains the most fundamental enhancement approach, effectively reducing random noise induced by laser intensity fluctuations and other environmental perturbations [11,12].

In signal optimization, coded excitation techniques have demonstrated unique advantages. Mienkina et al. developed a multispectral coded excitation system based on orthogonal unipolar Golay codes (OUGC) in 2009. By overcoming the limitations of conventional pulse repetition frequencies, their system achieved a coding gain of 9.8 dB with a 512-bit sequence. Moreover, this approach enabled simultaneous dual-wavelength PA acquisition and yielded significant SNR improvement (6.0−30.1 dB gain) at a 3 cm detection depth compared with traditional averaging [137,138].

Following preprocessing, multivariate statistical analysis methods form the backbone of glucose prediction modeling. Partial least squares regression (PLSR) and principal component analysis (PCA) are widely adopted due to their robustness against collinearity, effectively reducing the dimensionality of multi-wavelength PA data [111]. However, traditional linear models often struggle to account for the complex, non-linear dependencies arising from tissue heterogeneity and environmental fluctuations.

More recently, advanced machine learning algorithms have demonstrated superior modeling capabilities by serving as sophisticated non-linear filters and high-dimensional regressors. For instance, artificial neural networks (ANNs) and deep learning architectures enable intricate non-linear mapping through deep feature extraction, which is particularly effective in suppressing the background interference caused by fluctuating physiological parameters [106,133]. Support vector machines utilize kernel functions to project PA signals into higher-dimensional spaces, excelling in high-accuracy regression even in small-sample clinical scenarios [109]. Furthermore, ensemble learning methods like random forests and gradient boosting decision trees (GBDTs) offer enhanced interpretability by identifying the most significant PA spectral features, thereby facilitating the exclusion of wavelength-specific noise [125]. By integrating these ML models, the system can adaptively compensate for the “cross-sensitivity” of interfering components (e.g., water, lipids, and proteins) that overlap with the glucose PA spectrum. It should be emphasized, however, that all predictive models must be strictly calibrated and cross-validated against invasive glucose meters or laboratory reference methods [127], a process essential for ensuring clinical reliability and long-term stability in diverse user populations.

In summary, this section has systematically reviewed the core architecture and performance optimization strategies of PA glucose detection systems—from laser source selection to transducer, analog front-end, and quantizer performance requirements, and finally to the major interference mechanisms and their suppression approaches in practical measurements. A summary Table A2 is provided in Appendix B, consolidating the system configurations and key parameters of representative PA glucose studies, enabling readers to quickly compare design methodologies and application scenarios. This synthesis also lays the foundation for exploring future improvements in system performance and miniaturization.

## 4. Future Perspectives: Toward Wearable and Miniaturized Systems

Building on the preceding systematic review, this paper has outlined the fundamental principles, system architecture, and key performance indicators of PA-based glucose monitoring while identifying the primary factors influencing accuracy and stability. A retrospective view of its development reveals a clear trajectory toward higher integration, improved sensitivity, and enhanced robustness. However, it must be acknowledged that the vast majority of current PA glucose sensing research remains in the “lab table-top” stage. Despite promising results, there is a notable scarcity of recently published works that include independent clinical validations or large-scale human trials, which are essential prerequisites before transitioning to portable platforms.

To bridge the gap from laboratory prototypes to practical deployment, the field must address the significant technological gaps existing between benchtop setups and wearable form factors. This transition is primarily driven by four critical dimensions: (1) the replacement of bulky solid-state lasers with energy-efficient Pulsed Laser Diodes (PLDs) and integrated drivers; (2) the development of flexible, CMOS-compatible ultrasound transducers; (3) the transition from benchtop digitizers to low-power Application-Specific Integrated Circuits (ASICs); and (4) the shift toward intelligent algorithms for multimodal data fusion.

**Figure 11:**
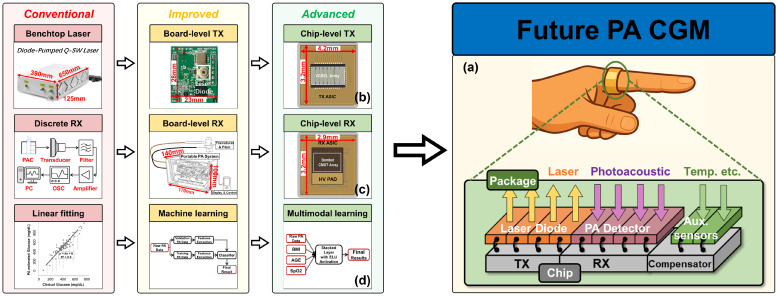
Conceptual illustration of future wearable PA glucose monitoring devices: (**a**) CGM wearable device with all-in-chip fully integrated PA sensors; (**b**) miniaturization of the laser source from OPO [139] to Board-level pulsed laser source [140], and ultimately to Chip-level integrated PA TX, reproduced from [141]; (**c**) miniaturization of the PA RX from discrete instruments [80] to Board-level RX, reproduced from [65], and ultimately to a monolithically PA ASIC-CMUT SoC, reproduced from [49]; (**d**) Algorithm-powered glucose assessment model from simple linear fitting to machine learning-based glucose prediction [106], and ultimately to multimodal learning, reproduced from [118].

With continuous advancements in these four domains, the realization of a truly wearable and continuous PA glucose monitoring system, as illustrated in Figure 11a, is steadily transitioning from concept to reality, positioning itself as a strong candidate for next-generation noninvasive glucose monitoring devices. This section focuses on the key technological trends driving the wearability of PA systems and highlights recent breakthroughs and frontier developments that are propelling the field toward miniaturization and practical deployment across the following four subsections.

### 4.1. Miniaturization of Laser Source: Advanced High-Energy Pulsed Laser Diodes and Driver Chips

Benefiting from the rapid progress in semiconductor laser technology and micro–nano integration packaging, recent research on PA glucose monitoring has increasingly adopted compact and PLDs as excitation sources. These lasers are capable of delivering narrow-pulse, high-peak-power emissions at specific wavelengths, particularly high-power PLDs operating in the near-infrared window, which can effectively induce PA responses in target tissues while significantly reducing system size and power consumption [65,108,109]. At the same time, the continuous optimization of laser driver boards [140] or even chips have provided strong support for system integration. Modern high-performance driver chips not only enable precise pulse-width control but also support multi-channel synchronization and high-current driving, thereby meeting the requirements of multi-wavelength and multi-channel PA excitation [142,143]. In addition, the maturation of chip-level LD packaging technology has greatly reduced parasitic inductance introduced by packaging, fully unleashing LD performance while achieving high-level integration of laser sources, power management, and thermal control, thereby enhancing overall system reliability and compactness [141,142]. Current LDs typically support nanosecond-scale pulse-width modulation and feature excellent thermal stability and long operational lifetimes. For example, edge-emitting lasers (EELs) and VCSELs, with their high cavity Q-factors, low threshold currents, and circular beam profiles that facilitate optical coupling, have emerged as ideal light sources for miniaturized PA systems [144,145,146]. Therefore, as illustrated in Figure 11b, the trend toward miniaturization and high integration of laser sources has become a core driving force for advancing PA-based glucose monitoring devices toward practical and wearable applications. This evolution not only reduces system size and power consumption but also enhances detection efficiency and sensitivity, providing essential support for the development of future portable PA glucose monitoring systems.

### 4.2. Miniaturization of Ultrasound Transducer: Flexible, High-Sensitivity Transducers Compatible with CMOS Processes

On the receiving side of PA glucose monitoring systems, ultrasound transducers are undergoing a continuous evolution toward higher sensitivity, miniaturization, and seamless integration. Early setups primarily employed PZT transducers, which offered simple structures and high sensitivity but suffered from bulky form factors and limited compatibility with electronic integration. In recent years, microelectromechanical system (MEMS)-based transducers have gained increasing attention. CMUT, which detects ultrasound via capacitance variations between a movable membrane and a fixed electrode, provides wide bandwidth and high sensitivity while enabling monolithic integration with CMOS circuitry on the same substrate. PMUT fabricated using thin-film piezoelectric materials operates at lower driving voltages and offers greater potential for miniaturization [147]. These emerging devices can rival traditional piezoelectric elements in performance, and with advances in CMOS compatibility, transducer arrays can now be monolithically integrated with readout circuits, effectively reducing noise from signal transmission paths and lowering packaging complexity [48,49]. Furthermore, the advent of flexible substrates and stretchable electronics allows ultrasound transducers to conform to complex surfaces, such as human skin or organ interfaces, with several wearable prototypes already demonstrating technical feasibility [47]. Looking ahead, flexible, high-sensitivity, and process-compatible transducers hold strong potential to merge with PA glucose detection, making them ideal candidates for clinically viable, next-generation wearable PA systems.

### 4.3. Miniaturization of Data Acquisition System: Application-Specific Integrated Circuit Design for Photoacoustic Detection

In recent years, efforts to reduce the size and cost of PA receivers have driven a transition from discrete components to board-level integration, with ASICs tailored to PA signal characteristics emerging as a research hotspot [148,149]. By customizing AFE, ASICs can achieve high-fidelity acquisition and processing of weak PA signals under low-power conditions. For example, as shown in Figure 11c, the Robert Bosch team developed a fully integrated PA signal detection system-on-chip (SoC), fabricated using the ST-28-FD-SOI process, which monolithically integrates AFE with CMUTs. The chip further employs a third-order delta–sigma modulator (DSM) to enable high-resolution quantization, achieving a signal chain dynamic range of up to 90 dB, thereby providing an important reference for chip-level PA receiver design [49]. Building on this foundation, a variety of signal-enhancement techniques—including coherent detection, early-and-late tracking, and adaptive bandwidth tuning—have been introduced into PA ASICs to further enhance detection capabilities [65,66,81,82,83,84,85]. Overall, the miniaturization of PA sensing is steadily advancing toward high sensitivity, low power consumption, and system-level integration. However, most existing ASIC implementations remain focused on PA imaging and temperature monitoring applications, with no dedicated circuits yet reported for PA-based glucose monitoring. Future work should address the unique signal characteristics and practical requirements of glucose detection by proposing more application-specific circuit architectures and SoC designs. Achieving high-fidelity extraction and robust processing of PA signals at the chip level will not only help overcome challenges of stability and inter-individual variability but also provide a solid technological foundation for realizing wearable PA glucose monitoring devices.

### 4.4. Enhanced Robustness and Generalizability: Algorithmic Empowerment and Multimodal Data Fusion

In the early stages of research, owing to the favorable linear relationship observed between PA signal features such as peak-to-peak amplitude and time-to-peak with glucose concentration, researchers primarily adopted linear regression and least-squares regression for modeling [59,97,117,122,125]. However, as research transitioned toward practical, out-of-laboratory applications, simple linear approaches proved insufficient in addressing the multi-source interferences arising from complex in vivoenvironments and dynamic physiological states. Consequently, advanced multivariate analysis and machine learning algorithms have been increasingly integrated into PA platforms to enhance system-level reliability. These include polynomial fitting [75,130], principal component regression (PCR) [107,111], support vector machines (SVMs) [109,125], random forests [125], and kernel-based methods [77,117]. By projecting low-SNR signals into high-dimensional feature spaces, these algorithms demonstrate notable improvements in model fitting and generalization across diverse user populations.

The rapid advancement of neural networks has further empowered PA sensing with unprecedented predictive accuracy and adaptive capabilities. Beyond traditional backpropagation neural networks (BPNNs) [121,134], specialized architectures such as convolutional neural networks (CNNs) for spatial-temporal feature learning [64,87] and wavelet neural networks (WNNs) for multiscale signal decomposition [86] have shown superior robustness against motion artifacts and baseline drifts−challenges that are particularly acute in wearable scenarios. These deep learning frameworks excel at capturing latent physiological correlations, effectively compensating for individual variations in skin thickness, melanin content, and hydration levels. Furthermore, multimodal fusion−as illustrated in Figure 11—has emerged as a decisive trend to address the inherent vulnerability of single-source PA signals to environmental fluctuations. For instance, integrating multidimensional physiological parameters [118], such as skin temperature and heart rate, allows for complementary information at both the molecular and systemic levels to be synthesized. Hybrid sensing architectures combining PA glucose monitoring with optical rotation sensing [67] or photoacoustic imaging (PAI) for structural−functional co-registration [63,120] significantly broaden the system’s applicability. Looking ahead, the synergy of algorithmic empowerment and multimodal data fusion, supported by efficient edge-computing hardware, is expected to drive PA-based glucose sensing toward long-term autonomous reliability, adaptive personalization, and seamless integration into pervasive wearable healthcare ecosystems.

In summary, the wearability of PA glucose monitoring systems depends not only on breakthroughs within individual modules but also on synergistic optimization across functional components. From the miniaturization of lasers with high pulse efficiency to the integrated design of novel ultrasound transducers, and further to the system-level integration of low-power dedicated chips, each technological advancement lays the foundation for building portable, low-power, and highly stable wearable systems. Meanwhile, the incorporation of artificial intelligence and multimodal fusion techniques offers new strategies to address signal fluctuations and inter-individual variability under complex real-world conditions. Looking forward, only by continuously fostering the coordinated development of all system modules while exploring power management, mechanical flexibility, and user-friendliness tailored for wearable applications can the transition from “laboratory prototypes” to “everyday applications” be truly achieved.

## 5. Conclusions

This review systematically outlines the development trajectory and fundamental principles of photoacoustic glucose monitoring technology, providing a comprehensive overview of recent methodological advances. Particular emphasis is placed on the design concepts and optimization strategies of key system components. For critical parameter selection, quantitative analyses are presented, along with a summary of the interference mechanisms in the glucose–photoacoustic response and corresponding suppression methods, as well as signal processing techniques for SNR enhancement in real-world applications. From a system integration perspective, advanced pulsed laser diodes with dedicated driver chips, process-compatible high-sensitivity transducers, photoacoustic-specific integrated circuits, and algorithm-enabled multimodal fusion approaches are offering new solutions to challenges in miniaturization, adaptation to individual variability, and long-term reliability. Collectively, these advances are accelerating the transition of photoacoustic noninvasive glucose monitoring from laboratory research to clinical and wearable applications. By integrating optics, electronics, materials science, and AI, this technology promises high precision, low power consumption, cost-effectiveness, and broad accessibility—ushering in a transformative era in diabetes management.

## Figures and Tables

**Table 1:** Guidance for SMBG.

Group	Recommended SMBG Frequency	Test Time	Reference
T1DM	150–200 monthly	Before/after meals, bedtime, **nighttime**	[6]
GDM	150–200 monthly	Fasting + 1 h/2 h post-meal; **nighttime** if needed	
T2DM-Intensive Insulin Therapy	100–150 monthly	Fasting, before/after meals, **bedtime** if needed	
T2DM-Basal Insulin	50 monthly	Fasting, before dinner	
T2DM-Tablet Medication	50 monthly	Fasting, after meals; and when ill or low BG	
T2DM-Diet & Lifestyle Mgmt.	2–3 weekly	Fasting, before meals, and 2 h after meals	
High-Risk Non-Diabetes	Once a year	Fasting & 75 g OGTT	[7]
Healthy	Every 3 years	Fasting & 75 g OGTT	

**Table 2:** Acoustic properties of selected typical materials [114].

Material	ρ (kg/$m^{3}$)	v (m/s)	Acoustic Impedance (1 × $10^{6}$·kg/(s·$m^{2}$))
Air	1.3	330	0.00429
Water	1000	1450	1.45
Muscle	1075	1590	1.70
Aluminum	2700	6320	17.1
Iron	7700	5900	45.43
Steel	7800	5900	46.02
Gold	19,320	3240	62.6
Skin	1109	1540	1.6

## Data Availability

The data used and/or analyzed during the current study are available from the corresponding author on reasonable request.

## Appendix A

**Table A1:** Comparison of different glucose monitoring technologies.

Terms	IDEAL	SMBG	CGM	RS	IS	MHC	PA
Invasive-ness	Non.	Inv.	Mini.	Non.	Non.	Non.	Non.
Time Lag	No	No	5~15 min [150]	Measurement in aqueous humor or dermis with low time lag	Measurement in dermis with low time lag [35]	Indirect measurement, lacking research on time lag	Measurement in the vascularized dermis, lower delay
Cost	Low	0.3$~1.0$ per test [151]	88$~107$ per sensor [151]	Raman spectrometers have high cost, yet nearly zero cost per use [27]	Main cost lies in optical detection components, nearly zero cost per use	Optical and thermal sensors have low cost, nearly zero cost per use	Main cost lies in laser source and transducer, nearly zero cost per use
Accuracy	Perfect	MARD ~5% [150]	MARD ~10% [152]	MARD ~14.6%, CEG A+B: 99.4% [27]	LOD: 10 mg/dL [92]	Susceptible to environmental interference, requires frequent calibration [34]	Near clinical standard, CEG A + B: <99% [153]
Stability	Perfect	Out-standing, often used as a reference standard	<14 days [17]	Periodic calibration of spectral equipment is required [27]	Sensitive to interfering substances, requiring frequent calibration [92]	Poor long-term stability [34]	Skin differences and environmental interference necessitate calibration [64]
Power	No	Several months to years	<14 days [17]	High power consumption due to spectrometers and high-power lasers	LED and detection circuit enable mW-level power consumption and battery-powered operation [92]	PPG, temperature and humidity measurement with low power consumption [33]	Depends on laser sources and receivers. Relatively high power consumption
Mini. Pot.	Wearable	Handheld device	Wearable	Limited by Raman spectrometers and high-energy laser sources, miniaturization is ongoing [154]	Mature sensors, and chip-scale spectrometers enhance miniaturization potential [92,155]	Mature sensors, high integrability	Lasers, transducers and receivers show a trend toward integration [48,85,146]
Pen. Depth	Interact with Blood	Interact with Blood	Interact with ISF	Interact with ISF/Blood	<3 mm (NIR). [156]	Measurement of external environmental parameters, non-penetrating	Less optical scattering, deeper penetration depth, <4 mm (NIR) [90]

## Appendix B

**Table A2:** Summary of system parameters in representative photoacoustic blood glucose monitoring systems.

Ref	Type	Sample Range	Test Acc.	Light Source					Transducer			AFE			DAQ		
				Type	λ	PW	Eng.	PRF	Type	Freq	BW	Type	BW	Gain	Feat	Type	Rate
[157]	Pulsed NIR/Vis	Skin Phant (0–5 k); Pig blood (0–880)	$R^{2}$: 0.9747; $R^{2}$: 0.9976;	Q-switched Nd:YAG	532 nm; 1064 nm	≈10 ns	1 μJ; 2 μJ	200	PVDF; PZT	NA	NA	Preamp; 3-stage	3M	40– 60 dB	P-to-P	Dig. Osc.	100 M
[153]	Pulsed NIR-PA	Finger	CEG A: 67.86%; B: 31.12%; D: 1.02%	NIR-pulsed laser diode	905 nm; 1064 nm	100 ns	10 μJ	100	PZT	5 M	2.5–7.5 M	LNA	0–12 M	20 dB	P-to-P	Dig. Osc.	200 M
[79]	Pulsed NIR-PA	Gluc. aq. sol. (0–350 mg/dL)	RMSE: 24.21; RMSE: 0.3909	NA	1600 nm	7 ns	30 mJ/${\mathrm{cm}}^{2}$	NA	PZT	1 M	Frac. 75%	LNA	NA	54 dB	P-to-P; T-to-P	Dig. Osc.	100 M
[77]	Pulsed NIR-PA	Forefinger	RMSE: 19.46; MARD: 7.01%; CEG A: 89.9%	NIR-pulsed laser diode	905 nm; 1550 nm	50 ns	50 W	0.1–3 k	PZT	5 M	2.5–7.5 M	LNA	0–12 M	60 dB	Amp- based	Dig. Osc.	200 M
[63]	Pulsed MIR-PA	Volar hypothenar	MARD: 9.94%; MAD: 8.27 mg/dL	EC-QCL	950 ${\mathrm{cm}}^{-1}$ 1240 ${\mathrm{cm}}^{-1}$	500 ns	44.2 nJ	47.5 k	mic.	NA	NA	Lock-in amp.	30 ms	NA	P-to-P	DAQ	NA
[74]	CW NIR- PA	Left earlobe	R: 0.84; S.E.: 21 mg/dL	DFB-NIR-LD	1382 nm; 1610 nm	NA	8 mW; 5–7 mW	NA	PZT	NA	NA	Lock-in amp.	NA	NA	Optical pwr bal. point	NA	NA
[105]	Pulsed NIR-PA	Gluc. aq. sol. (0–500 mg/dL)	$R^{2}>0.912$; RMSE: 22.4	Tunable OPO pulsed laser	1064 nm	8 ns	2 mJ	20	PZT	2.5 M	NA	Preamp.	50 k–40 M	40 dB	P-to-P; T-to-P	Dig. Osc.	500 M
[111]	Pulsed NIR-PA	Human serum	$R^{2}$: 0.995; CEG A: 100%	Tunable OPO pulsed laser	1600 nm	9 ns	20 mJ/${\mathrm{cm}}^{2}$	20	PZT	2 M	60%	Preamp.	50 k–2 M	54 dB	Time domain features	Dig. Osc.	100 M
[106]	Pulsed MIR-PA	Skin phantom	Acc: ±25 mg/dL; CEG A: 100%	QCL	1080 ${\mathrm{cm}}^{-1}$	30 μs– 100 μs	60 W	10–30 k	mic.	15– 30 kHz	NA	Lock-in amp.	30 ms	NA	Peak voltage	NA	NA
[80]	Pulsed NIR-PA	Gluc. aq. sol. (0–320 mg/dL)	$R^{2}$: 0.8602; CEG A: 71%; CEG B: 29%	NIR-pulsed laser diode	1535 nm	4 ns	365 ± 2 μJ	4	PZT	2.25 M	NA	Preamp.	NA	30 dB	Peak; Wave	Dig. Osc.	NA
[125]	CW NIR- PA	Gluc. aq. sol. (40–400 mg/dL)	RMSE: 13.97; CEG: 92.38%	Low power CW-laser	1500 nm– 1630 nm	1/434 k	40 mJ/${\mathrm{cm}}^{2}$	434 k	PZT	500 k	NA	Lock-in amp.	10 ms	NA	Peak voltage	NA	NA
[67]	Pulsed NIR-PA	Serum sample	LOD: 80 mg/dL	Tunable OPO pulsed laser	1560 nm	7 ns	2.5 mJ; 1.18 mJ	30	PZT	7.5 M	60%	Preamp.	550 k– 30 M	30 dB	PA signal analysis	Dig. Osc.	1 G
[118]	Pulsed NIR-PA	Finger	RMSE: 2.86; MAD: 8.77; MARD: 8.49%	NIR-pulsed laser diode	905 nm	100 ns	10 μJ	100	PZT	NA	NA	LNA	6.5 M	40– 60 dB	P-to-P	Mix-sig. Osc.	200 M
